# Determination of UDP-Glucose and UDP-Galactose in Maize by Hydrophilic Interaction Liquid Chromatography and Tandem Mass Spectrometry

**DOI:** 10.1155/2022/7015311

**Published:** 2022-06-28

**Authors:** Chen Lan, Bing Zhao, Lu Yang, Yusen Zhou, Siyi Guo, Xuebin Zhang, Junli Zhang

**Affiliations:** State Key Laboratory of Crop Stress Adaptation and Improvement, School of Life Sciences, Henan University, Kaifeng 475004, China

## Abstract

Nucleotide sugars, the activated forms of monosaccharides, are important intermediates of carbohydrate metabolism in all organisms. Here, we describe a method for the detection and quantification of UDP-glucose and UDP-galactose in maize in order to compare their metabolism in both wild-type and mutated plants. Triple quadrupole operating in a multiple reaction monitoring mode was used to quantify nucleotide sugars. The limits of detection for UDP-glucose and UDP-galactose were 0.50 and 0.70 ng·mL^−1^, respectively. The recoveries of the method ranged from 98.3% to 103.6% with the relative standard deviations less than 6.3%. To prove the applicability of this method, we analyzed several sets of maize extracts obtained from different cultivars grown under standardized greenhouse conditions. All the results demonstrated the suitability of the developed method to quantify UDP-glucose and UDP-galactose in maize extracts.

## 1. Introduction

Carbohydrates are widespread in nature, just as proteins and nucleic acids are the essential macromolecular substances of living organisms [1]. In living organisms, carbohydrates are present in different forms, including glycans, glycoproteins, proteoglycans, glycolipids, and so on. Carbohydrates serve as a major carbon source for the growth of viruses, bacteria, fungi, plants, and animals. In addition, carbohydrates play diverse roles in important physiological processes, such as cell structure composition, cell differentiation, cell surface recognition, signal transduction, cellular immunity, and pathogen invasion [2–4]. Hence, the quantitative determination of carbohydrates is of great significance.

Nucleotide sugars, the main precursors for glycan synthesis, are activated sugar donors. They are involved in a multitude of cellular processes in plants, such as the interconversion of sucrose and starch, biosynthesis of cell wall carbohydrate polymers, and metabolic regulatory processes [5–7]. There are two major routes for nucleotide sugars synthesis in plants: the de novo and salvage pathways [8, 9]. Uridine 5′-diphosphate (UDP)-glucose is one of the most important members among these nucleotide sugars and plays a central role in the interconversion of energized sugars. For example, UDP-glucose is the precursor to UDP-galactose, UDP-rhamnose, and UDP-glucuronic acid, an essential component of the cell wall [5].

A specific, sensitive, and robust quantification method is required to better understand the functionality of nucleotide sugars. Owing to their similar structures, it is challenging to achieve complete chromatographic separation. On one hand, UDP-galactose and UDP-glucose differs only by the orientation of a hydroxyl (-OH) group. On the other hand, they are present at low levels in plant samples [5]. To achieve highly sensitive and accurate quantification of UDP-galactose and UDP-glucose, researchers have been developing many chromatographic methods, such as ion-exchange chromatography [6, 10], ion-pair chromatography [5, 9, 11], capillary electrophoresis [7, 12], reversed phase liquid chromatography [7], liquid chromatography coupled with mass spectrometry [13], and so on. Ion-exchange chromatography can be used to separate UDP-galactose and UDP-glucose. The main problem of ion-exchange chromatography is too high salt concentration and it is incompatibility of the used nonvolatile salt for mass spectrometry. Traditionally, reversed phase liquid chromatography is used to detect apolar or slightly polar molecules [14]. Therefore, it is hardly suited to separate highly polar nucleotide sugars.

LC-MS becomes an increasingly important technique for the separation of nucleotide sugars. Zhou et al. used a bare titania column to separate nucleotides and their intermediates including UDP-uridine 5′-diphospho-N-acetyl-D-glucosamine (UDP-GluNAc) and UDP-glucose [15]. Behmüller et al. developed a HPLC-ESI-MS/MS method based on a porous graphitic carbon (PGC) column to separate UDP-sugars in plant cells [16]. Nonetheless, they reported issues with retention time instabilities. This problem was overcome by grounding of the column effluent and column regeneration procedure. Ito et al. published data in which they used hydrophilic interaction chromatography (HILIC) coupled with a triple quadrupole mass spectrometer on a ZIC-pHILIC column, but they were unable to separate the isomers UDP-galactose and UDP-glucose [13]. Warth et al. successfully separated and analyzed UDP-glucose by using the zwitterionic stationary phase-based chromatography utilizing mass spectrometric detection [17].

For polar compounds, it is hardly retained in RPLC. HILIC methods have the potential to retain and separate polar metabolites that show no retention or coelute in RPLC and can lead to an increased MS sensitivity for polar compounds [18–20]. HILIC uses an aqueous-organic mobile phase, which is a special subtype of normal-phase liquid chromatography [21, 22]. A polar stationary phase is used in combination with an aqueous-organic mobile phase, which creates a water-rich layer around the stationary phase, in which various hydrophilic interaction mechanisms occur, such as hydrogen bonding, electrostatic interactions, and dipolar interactions. Due to their polar nature, they are weakly retained on a reversed-phase HPLC column [21, 23–25].

Here, we present a simple, effective, and highly sensitive UPLC-ESI-MS/MS method through hydrophilic interaction using amide column coupled with a triple quadrupole operating in the multiple reaction monitoring mode to detect UDP-galactose and UDP-glucose. This method is simple, and grounding is no longer required, which greatly improves the problem of instable retention times. In addition, the configuration of UDP-glucose and galactose in the commercial standard may be different from those in plant samples. We developed a method to isolate and prepare these two sugars from plant samples, and it is convenient to further investigate the roles of sugars in plants for other researchers. Preparative RP-HPLC was used to isolate UV-vis quantifiable amounts of UDP-glucose and UDP-galactose from plant samples. This study provides a useful analytical method for studies of the level of UDP-glucose and UDP-galactose in maize.

## 2. Materials and Methods

### 2.1. Chemicals and Reagents

All chemicals were of analytical grade or higher and were used as received without any further purification. Deionized water was purified using a Milli-Q system from Millipore (Millipore, USA). HPLC-grade methanol and acetonitrile were purchased from Thermo Fisher Scientific (USA). Standards of UDP-glucose and UDP-galactose were purchased from Merck (Germany). Formic acid (99%, HPLC-grade) and ammonium formate (99%, LC-MS grade) were purchased from Merck (Germany). Potassium phosphate (KH_2_PO_4_) was purchased from Sigma. Sample vials, vial inlets, and vial snap caps were purchased from Merck (Germany). The HSS T3 column (2.1 × 100 mm, 1.8 *μ*m) and BEH amide column (2.1 × 100 mm, 1.7 *μ*m) were both obtained from Waters (UK).

### 2.2. Preparation of Analytical Standards

The standard solutions were prepared by dissolving solid standards in a combination of water with methanol. The solutions were stored at −20°C before injection.

### 2.3. Preparative HPLC

UDP-glucose and UDP-galactose were fractionated using RP-HPLC on a Waters 2998 HPLC device (Waters, Wilmslow, UK) equipped with an Agilent NH_2_ column (4.6 × 250 mm, 5 *μ*m particle size; Agilent, USA). Chromatographic elution was performed at a flow rate of 1 mL/min using a linear potassium phosphate buffer/methanol gradient. The mobile phases were composed of mixture of 80 mM potassium phosphate buffer at pH 3.6 as mobile phase A and methanol as mobile phase B (A : B, 40 : 60, v/v). Fractions (24.6 min and 24.8 min) were collected using a Waters Fraction Collector (Waters, Wilmslow, UK).

### 2.4. UPLC-MS/MS Method

Detection and quantification of analytes were conducted on a TQ-XS system (Waters, Wilmslow, UK) equipped with an electron spray ionization (ESI) source. It consists of a temperature-controlled column chamber, auto-sampler, and quaternary pump. For data acquisition and analysis, MassLynx V4.2 was used on the system in Microsoft Windows 10 environment.

The chromatographic separation was performed on an ACQUITY UPLC H-Class plus system (Waters, Wilmslow, UK) using a BEH amide column, 2.1 × 100 mm with 1.7 *μ*m particle size (Waters, Wilmslow, UK). The mobile phase consisted of 50 mM ammonium formate solution (pH = 3.6, A) and acetonitrile (B). The elution was carried out under the following conditions: 79% A : 21% B. The flow rate was set to 400 *μ*L/min, the injection volume was 5 *μ*L, and the temperature of the column was maintained at 30°C.

ESI-MS/MS was done in the negative-ion mode. The optimum conditions of multiple reaction monitoring (MRM) were carried out. Two individual transitions were monitored per analyte with the following settings: ion spray voltage, 3500 V; auxiliary gas pressure, 5 arb. units; ion transfer tube temperature, 350°C; ion source temperature, 150°C. The values of collision energy, cone voltage, and transitions for the MRM mode are given in Table 1.

### 2.5. Plant Material and Growth Conditions

Maize plants were grown 14-h light/10-h dark conditions at 28°C/20°C. The light intensity was 400 *μ*mol·m^2^·s^−1^, and the relative humidity was kept at 40%.

### 2.6. Sample Preparation

The plant material (during postripening stomatal development) was frozen in liquid nitrogen and ground carefully to fine powder with 5.5 mm stainless steel balls in a mortar. Samples were stored at −80°C.

The homogenized maize material (100 ± 2 mg) were weighed into Eppendorf tubes and extracted with 1 mL of water/methanol (25/75, v/v) including 0.1% formic acid, vortexed, and further treatment was done in an ultrasonic bath at room temperature for 15 min. Samples were centrifuged at 8500 × *g* for 10 min at 4°C. The samples were passed through a 0.22 *μ*m membrane, and 100 *μ*L of the supernatant was transferred into a glass vial.

### 2.7. Method Validation

Validation of the developed method was evaluated, and the parameters were investigated including linear range, limit of detection, limit of quantification, precision, recovery, selectivity, and sensitivity. Intraday and interday precision as well as the recovery of analytes were determined by measurements of a maize extract mixture spiked with the standard working solution at three different concentration levels.

## 3. Results and Discussion

### 3.1. UPLC-ESI-MS/MS Method Development

**Figure 1:**
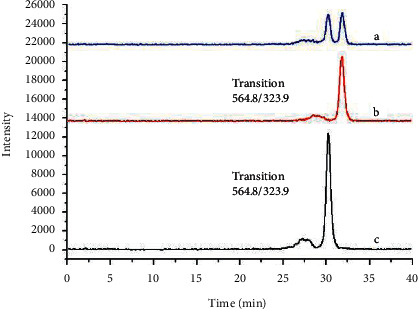
Liquid chromatography-tandem mass spectrometry chromatogram of standards. (a) Mix of UDP-galactose and UDP-glucose. (b) UDP-galactose. (c) UDP-glucose.

**Figure 2:**
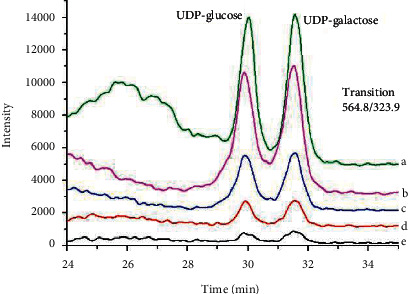
Liquid chromatography-tandem mass spectrometry chromatogram of different concentrations UDP-galactose and UDP-glucose. (a) 500 ng/mL, (b) 250 ng/mL, (c) 125 ng/mL, (d) 62.5 ng/mL, and (e) 31.25 ng/mL.

In order to achieve satisfactory separations and high responses for two target analytes, the separation parameters were optimized. First, two kinds of HPLC columns and the mobile phase were evaluated and optimized. Two kinds of columns were tested, including HSS T3 (2.1 mm × 100 mm, 1.8 *μ*m) and BEH amide (2.1 mm × 100 mm, 1.7 *μ*m). For T3 column, we were unable to separate the two target analytes. As we can see from Figure 1, they could be completely separated on the column of BEH amide. Eluent composition was optimized for the separation and sensitive determination of target compounds. During the method development, the effect of the organic content on the mobile phase, the effect of ammonium formate concentrations, and the effect of buffer pH were investigated. When the acetonitrile/water or acetonitrile/0.01% formic acid were used as the mobile phase, the chromatographic separation of the structural isomers UDP-glucose and UDP-galactose was not successful. The results revealed that we changed the aqueous phase to 50 mM ammonium formate and found that the two analytes were successfully separated, and the tailing of the target peak was reduced. Longer retention times can be explained by the lower eluotropic strength of acetonitrile compared to water. When we changed the organic content from 79% to 78%, the retention time became shorter, but the resolution of the analytes was decreased as well. The best separation effects with high responses were achieved using the conditions described in Section 2.4. There was no shift in retention time during the run time over four hours (as shown in Figure 2). Compared to other methods, the development method greatly improved the problem of instable retention time.

For two analytes to yield two specific transitions for specific transitions for qualification and quantification for each analyte in negative ionization mode, automated optimization of tuning parameters by means of the MassLynx V4.2 software was carried out. Usually two product ions with the highest sensitivity were selected. A chromatogram of reference standards was displayed in Figure 1. The optimal cone voltage and collision energy for the parent ion and daughter ion are listed in Table 1.

### 3.2. Method Validation

**Figure 3:**
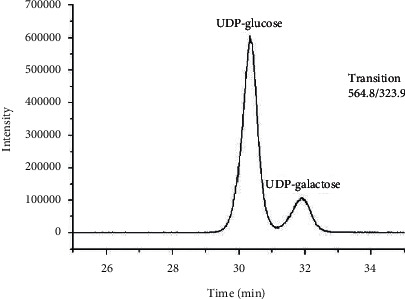
Liquid chromatography-tandem mass spectrometry chromatogram of maize samples (E).

To investigate the suitability and practicability of this method, a series of parameters, including linear range, intraday and interday precision, selectivity, matrix effect, LOD and LOQ values, were validated. Under the above optimized conditions, the method validation parameters are presented in Tables 2 and 3. The LC-ESI-MS/MS chromatogram of the maize sample is shown in Figure 3.

The method showed good linearity over the concentration range from 31.25 ng/mL to 500 ng/mL for UDP-galactose and UDP-glucose. And the coefficients of determination were above 0.99 for UDP-galactose and UDP-glucose. The limit of detection (LOD) values for UDP-glucose and UDP-galactose were 0.50 and 0.70 ng·mL^−1^, respectively, based on a signal-to-noise (*s*/*n*) ratio of 3. And the LOQs were determined by an *s*/*n* equal to 10. The precision of the method was evaluated by analyzing the spiked products at three concentrations levels (as shown in Table 3), and each solution was measured in triplicate. As can be seen from Table 3, the average recoveries of UDP-galactose and UDP-glucose ranged from 98.3% to 103.6% with a max relative standard deviation (RSD) value of 6.3%. The intraday RSD was determined by analyzing six replicates on the same day, and the interday RSD was evaluated by analyzing three replicates in three different days. And the intraday or interday precision was appraised by RSDs (below 4.3%) of peak areas (*n* = 5) at 62.5 ng/mL. And the matrix effect was examined by comparing the slope of the calibration curves for the solvent and that of the ones obtained from the maize matrix extract (as shown in Table 2). These results suggested that the developed method was accurate and reproducible.

### 3.3. Application of the Method to Maize Samples

To demonstrate the applicability of the method developed in this study, we applied the established UPLC-ESI-MS/MS method for the determination of residual contents of UDP-galactose and UDP-glucose in maize samples in order to compare their metabolism both in wild-type and mutated plants.

The results of UDP-galactose and UDP-glucose in maize samples are shown in Table 4.

### 3.4. Comparison of the Proposed Method with Previously Reported Results

The performance of the developed UPLC-ESI-MS/MS method was compared with some other reported methods for the analysis of UDP-galactose and UDP-glucose in plant samples.

Compared with the published method (as shown in Table 5), all the results indicated that the proposed UPLC-ESI-MS/MS method in this work exhibited lower LODs and higher recoveries.

## 4. Conclusions

A simple, effective, and sensitive UPLC-ESI-MS/MS method for the quantification of UDP-galactose and UDP-glucose in the maize sample was successfully developed. UPLC-ESI-MS/MS demonstrated a wide dynamic range, good linearity, and sufficient sensitivity to quantitate these UDP-sugars in the maize sample. The method was validated for its use to analyze maize samples, but it might be applied for the analysis of other plant extracts due to the simple sample preparation protocol. The practical application of the new method was demonstrated by the determination of maize samples.

## Figures and Tables

**Table 1:** The MS parameters of UDP-glucose and UDP-galactose.

Analytes	RT (min)	ESI mode	Parent ion (*m*/*z*)	Daughter ion (*m*/*z*)	Cone voltage (V)	Collision energy (eV)
UDP-glucose	30.10	ESI-	564.8	78.89/322.9	100	46/22
UDP-galactose	31.89	ESI-	564.8	78.89/322.9	100	60/24

**Table 2:** Calibration curves and LOD and LOQ of UDP-galactose and UDP-glucose.

Analytes	Linear equation	Correlation coefficient (*r*^2^)	Limit of detection (ng·mL^−1^)	Limit of quantification (ng·mL^−1^)	Matrix effect (%)
UDP-glucose	*y* = 5.83231*x* − 60.3490	0.9982	0.50	1.67	11.75
UDP-galactose	*y* = 4.22794*x* + 25.4917	0.9991	0.70	2.33	15.63

**Table 3:** Precisions and recoveries of UDP-galactose and UDP-glucose.

Analyte	Precision (%)		Low ((%), 31.25 ng·mL^−1^)		Medium ((%), 125 ng·mL^−1^)		High ((%), 500 ng·mL^−1^)	
	Intraday *n* = 5	Interday *n* = 5	Recovery	RSD	Recovery	RSD	Recovery	RSD
UDP-glucose	1.7	2.6	103.6	5.6	98.3	2.7	100.3	2.8
UDP-galactose	2.9	4.3	99.6	6.3	101.7	5.9	101.5	4.9

**Table 4:** Results of UDP-galactose and UDP-glucose in different maize samples.

Sample	Analyte	Found (ng·g^−1^)	RSD (%), *n* = 6
WT	UDP-glucose	495451.33	5.44
	UDP-galactose	25982.33	7.80
bzu3-2	UDP-glucose	294219.00	12.90
	UDP-galactose	53782.67	9.14
Z58	UDP-glucose	1140085.75	7.66
	UDP-galactose	38516.25	7.02
ZE	UDP-glucose	250784.33	4.35
	UDP-galactose	32213.33	4.15
E	UDP-glucose	144199.33	9.18
	UDP-galactose	67730.00	8.40

**Table 5:** Comparison of different analytical methods for the determination of UDP-galactose and UDP-glucose in plant samples.

Sample	Analyte	Instrument technique	Limit of detection	Reference
*Arabidopsis thaliana*	UDP-glucose/UDP-galactose	HPLC-MS	70 nmol·L^−1^	[16]
Wheat	UDP-glucose	LC-ESI-MS/MS	1 ng·mL^−1^	[17]
Tobacco	UDP-glucose	LC-ESI-MS	5 nmol·L^−1^	[26]
Maize	UDP-glucose/UDP-galactose	UPLC-ESI-MS/MS	0.5 ng·mL^−1^/0.7 ng·mL^−1^	This work

## Data Availability

The data used to support the findings of this study are included within the article.
